# Chemical mutagenesis of *Caenorhabditis elegans* uncovers genetic modifiers of huntingtin protein aggregation

**DOI:** 10.17912/micropub.biology.000202

**Published:** 2020-01-02

**Authors:** Hailey M. Ung, Rhodes H. Hall, Elise A. Kikis

**Affiliations:** 1 Department of Biology, The University of the South, Sewanee, TN 37383

**Figure 1: Genetic modifiers of huntingtin protein aggregation in C. elegans.**
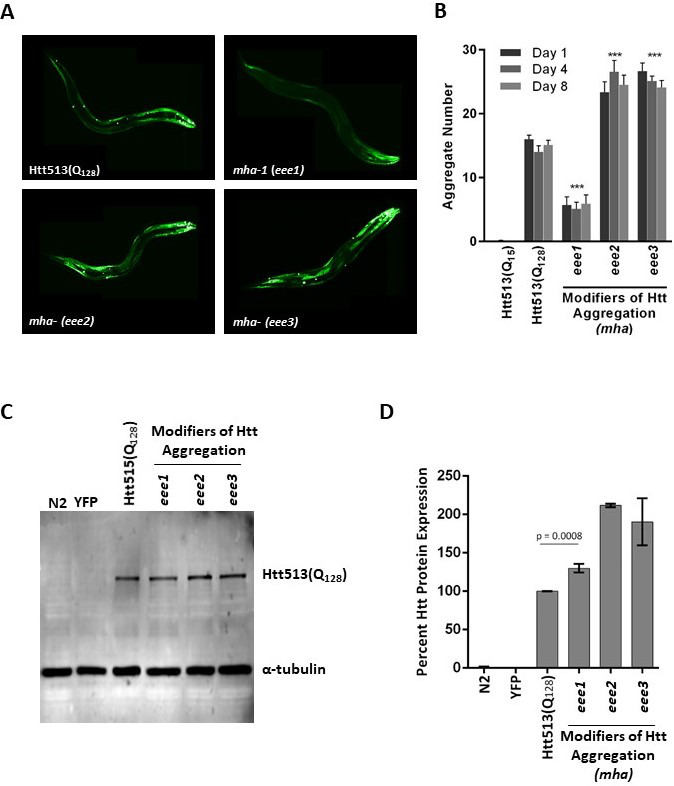
A) Representative fluorescent confocal micrographs of a parental strain (EAK103) expressing Htt513(Q_128_)::YFP in body wall muscle cells, or of three independent EMS-derived mutants referred to herein as *m*odifiers of *H*tt *a*ggregation (*mha*). B) Quantification of the number of Htt513(Q_128_)::YFP protein aggregates in each of the *mha* mutants compared to both the parental strain and to strain EAK102, which expresses the protein Htt513(Q_15_)::YFP as a negative control for aggregation. Mean aggregate number for at least 50 individuals is shown for each indicated time point. Animals were sacrificed at the time of scoring; therefore each day represents a completely independent experiment. Error bars represent standard error of the mean. Student t-tests were performed, comparing aggregation in each *mha* strain to that of the parental strain. Because no statistically significant differences were observed over time, data for each of the three days were pooled for the purpose of further statistical analyses. The symbol *** denotes a p-value less than 0.0001. C) Representative western blot of Htt513(Q_128_)::YFP protein detected with an anti-polyQ antibody or α-tubulin as a loading control. N2 and YFP-expressing animals (AM134) are shown as negative controls. D) Western blot quantification using Image J analysis software. Mean Htt protein levels are shown for three biological replicates, error bars represent standard error of the mean, and the p-value is the result of a student t-test.

## Description

Huntington’s disease (HD) is an autosomal dominant monogenic neurodegenerative disorder caused by a CAG trinucleotide repeat expansion in the gene encoding the protein huntingtin (Htt) (MacDonald et al., 1993). The resultant disease-associated Htt protein harbors a polyglutamine (polyQ) repeat that renders it metastable with respect to folding (Carrell and Lomas, 1997). Htt protein misfolding, characterized by the accumulation of misfolded protein aggregates and neurotoxicity, is first observed in mid- to late-life for most HD patients (Becher et al., 1998). The age-of-onset for HD is inversely proportional to CAG repeat length (Becher et al., 1998). Nonetheless, genetic variation between HD patients is attributed to slight differences in age-of-onset, even when repeat length is the same (Gusella and MacDonald, 2000). Thus, genetic background seems to be an important modifier of Htt protein aggregation and toxicity. We are interested in identifying genes/proteins that enhance or suppress the folding defect of human Htt.

To model Htt toxic-gain-of-function in the genetically tractable *Caenorhabditis elegans*, we previously characterized transgenic animals expressing a YFP-tagged polyQ-expanded disease-associated fragment of human Htt in *C. elegans* body wall muscle cells (Lee et al., 2017). More specifically, the first 513 amino acids of the human Htt protein were fused to YFP for visualization. Two different polyQ tract lengths (Q_15_ and Q_128_) were utilized, resulting in the proteins Htt513(Q_15_)::YFP and Htt513(Q_128_)::YFP, corresponding to the strains EAK102 and EAK103, respectively (Lee et al., 2017). For simplicity, these proteins are referred to herein as Htt513(Q_15_) or Htt513(Q_128_). As reported, only Htt513(Q_128_), not Htt513(Q_15_), formed protein aggregates in body wall muscle cells (Lee et al., 2017), consistent with only longer polyQ tracts being associated with disease.

Here, we describe the identification and characterization of genetic *m*odifiers of *H*tt *a*ggregation (*mha*). To this end, EAK103 animals expressing Htt513(Q_128_) were grown to the L4 larval stage and exposed to the alkylating agent ethyl methanesulfonate (EMS) at a final concentration of 50mM for 4hrs, according to established protocols (Brenner, 1974). In short, F1 individuals derived from the mutagenized parents were allowed to self-fertilize for one generation, yielding an F2 population, for the purpose of homozygosing recessive alleles and thereby uncovering mutant phenotypes. Screening of the F2 animals for those with increased or decreased aggregation was performed by eye with a fluorescent stereomicroscope.

Using this strategy, we obtained three independently-derived *mha* alleles, *mha-1*(*eee1), mha-(eee2),* and *mha-*(*eee3)* all displaying either increased or decreased Htt513(Q_128_) protein aggregation. Independence was assured by generating separate pools of F1 progeny with no more than one mutant individual selected from any given pool for further analysis. However, because allelism tests were not performed, we cannot say that our three alleles necessarily represent three different genes. Therefore, we are assigning only one allele, *eee1,* the specific gene name *mha-1*. Qualitatively, *mha-1(eee1)* displayed decreased Htt513(Q_128_) aggregation whereas *mha-(eee2)* and *mha-(eee3)* displayed increased aggregation (**Fig. 1A**). To determine the extent of aggregation suppression or enhancement, the number of Htt513(Q_128_) protein aggregates in each of the three *mha* mutant strains was quantified at days 1, 4, and 8 of adulthood and compared to that of the Htt513(Q_128_) parental strain (EAK103) and the Htt513(Q_15_) negative control strain (EAK102) (**Fig. 1B**). The aging time-course was to determine whether our new mutants had early or late effects or whether they worked in a synergistic manner with the aging program. We found that while the parental strain accumulated ~15 aggregates in body wall muscle cells on all days examined, *mha-1(eee1)* accumulated <10 aggregates, whereas *mha-(eee2)* and *mha-(eee3)* accumulated >20 aggregates. These numbers of aggregates were statistically different from the parental strain, but not affected by age in any of the mutants examined (**Fig. 1B**).

Because aggregation is a concentration-dependent phenomenon, we needed to rule out the trivial possibility that changes in aggregation were simply due to higher or lower transgene expression levels. To address this, we performed western blot analysis with an antibody raised against expanded polyQ or against α-tubulin as a loading control (**Fig. 1C**). Briefly, total protein from animals grown to day 1 of adulthood was extracted and loaded on a 10% SDS-polyacrylamide gel, transferred to a PVDF membrane, and incubated in the presence of the indicated antibodies. Visualization was with a Li-Cor Odyssey imaging system (Lincoln, NE). Quantification of protein levels from three independent experiments was performed with Image J. The analysis revealed that *mha-1(eee1)* accumulated more, not less, Htt513(Q_128_) protein than the parental control (**Fig. 1D**). This means that the underlying genetic lesion in *mha-1(eee1)* decreases aggregation without decreasing protein levels. In contrast, *mha-(eee2)* and *mha-(eee3)*, in which Htt513(Q_128_) aggregated more than the control, also accumulated more total protein. Thus, the observed increase in aggregation could be due to a higher concentration of available Htt513(Q_128_) protein. Alternatively, these aggregates themselves may be highly stable, such that increased aggregation may equate to less protein turnover, a longer half-life, and, consequently, higher steady-state protein levels.

Together, our data describe a screening strategy for the successful identification of genetic modifiers of Htt513(Q_128_) protein aggregation. Prior to this study, we were uncertain whether the long polyQ tract length and early aggregation of Htt513(Q_128_) in body wall muscle cells would render it impossible to suppress aggregation. However, *mha-1(eee1)* only forms a few aggregates in the muscles surrounding the head, but is otherwise completely diffuse. Thus, the mutants characterized herein are not only interesting in their own right as modifiers of protein aggregation, but they serve as a proof of principle, opening up the possibility for larger-scale studies in the future.

## Reagents

*C. elegans* were maintained on Nematode Growth Media (NGM) that was seeded with *E. coli* strain OP50 as a food source according to established protocols (Brenner 1974).

The *mha* mutant strains described herein are available by request. They are:

EAK104 *mha-1(eee1) eeeIs2*[P*unc-54*::Htt513(Q128)::YFP::*unc-54* 3’UTR]

EAK105 *mha-(eee2) eeeIs2*[P*unc-54*::Htt513(Q128)::YFP::*unc-54* 3’UTR]

EAK106 *mha-(eee3) eeeIs2*[P*unc-54*::Htt513(Q128)::YFP::*unc-54* 3’UTR]

The following previously published strains used in this study are available from the *C. elegans* Genetic Center (CGC):

N2 Wild type, Bristol, (Brenner 1974)

AM134 *rmIs126[*P*unc-54::Q0::YFP]* X, (Morley, Brignull et al. 2002)

EAK102 *eeeIs1*[P*unc-54*::Htt513(Q15)::YFP::*unc-54* 3’UTR], (Lee, Ung et al. 2017)

EAK103 *eeeIs2*[P*unc-54*::Htt513(Q128)::YFP::*unc-54* 3’UTR], (Lee, Ung et al. 2017)

For SDS-PAGE and western blot analysis, total protein from 10 nematodes was extracted directly into Laemmli sample buffer and loaded on a 10% SDS-PAGE gel. After electrophoresis, protein was transferred to an Immun-Blot Low Fluorescence PVDF membrane (Bio-Rad, Irvine, CA). The primary antibodies were the anti-expanded polyglutamines antibody 3B5H10 and the anti-alpha-tubulin antibody B-5-1-2, both from Millipore Sigma (Carlsbad, CA). The secondary antibody was the IRDye® 800CW Goat anti-Mouse IgG Secondary Antibody from Li-Cor (Lincoln, NE).
